# Combined impacts of environmental and socioeconomic covariates on HFMD risk in China: A spatiotemporal heterogeneous perspective

**DOI:** 10.1371/journal.pntd.0011286

**Published:** 2023-05-19

**Authors:** Chun-Hu Li, Jun-Jie Mao, You-Jia Wu, Bin Zhang, Xun Zhuang, Gang Qin, Hong-Mei Liu

**Affiliations:** 1 Joint Division of Clinical Epidemiology, Affiliated Hospital of Nantong University, School of Public Health of Nantong University, Nantong, China; 2 Department of Pediatrics, Affiliated Hospital of Nantong University, Nantong, China; 3 Department of Infectious Diseases, Affiliated Hospital of Nantong University, Nantong, China; 4 Department of Epidemiology and Biostatistics, School of Public Health of Nantong University, Nantong, China; 5 School of Transportation and Civil Engineering of Nantong University, Nantong, China; KU Leuven, Rega Institute, BELGIUM

## Abstract

**Background:**

Understanding geospatial impacts of multi-sourced influencing factors on the epidemic of hand-foot-and-mouth disease (HFMD) is of great significance for formulating disease control policies tailored to regional-specific needs, yet the knowledge is very limited. We aim to identify and further quantify the spatiotemporal heterogeneous effects of environmental and socioeconomic factors on HFMD dynamics.

**Methods:**

We collected monthly province-level HFMD incidence and related environmental and socioeconomic data in China during 2009–2018. Hierarchical Bayesian models were constructed to investigate the spatiotemporal relationships between regional HFMD and various covariates: linear and nonlinear effects for environmental covariates, and linear effects for socioeconomic covariates.

**Results:**

The spatiotemporal distribution of HFMD cases was highly heterogeneous, indicated by the Lorenz curves and the corresponding Gini indices. The peak time (R^2^ = 0.65, *P* = 0.009), annual amplitude (R^2^ = 0.94, *P*<0.001), and semi-annual periodicity contribution (R^2^ = 0.88, *P*<0.001) displayed marked latitudinal gradients in Central China region. The most likely cluster areas for HFMD were located in south China (Guangdong, Guangxi, Hunan, Hainan) from April 2013 to October 2017. The Bayesian models achieved the best predictive performance (R^2^ = 0.87, *P*<0.001). We found significant nonlinear associations between monthly average temperature, relative humidity, normalized difference vegetation index and HFMD transmission. Besides, population density (RR = 1.261; 95%CI, 1.169–1.353), birth rate (RR = 1.058; 95%CI, 1.025–1.090), real GDP per capita (RR = 1.163; 95%CI, 1.033–1.310) and school vacation (RR = 0.507; 95%CI, 0.459–0.559) were identified to have positive or negative effects on HFMD respectively. Our model could successfully predict months with HFMD outbreaks versus non-outbreaks in provinces of China from Jan 2009 to Dec 2018.

**Conclusions:**

Our study highlights the importance of refined spatial and temporal data, as well as environmental and socioeconomic information, on HFMD transmission dynamics. The spatiotemporal analysis framework may provide insights into adjusting regional interventions to local conditions and temporal variations in broader natural and social sciences.

## Author summary

Hand-foot-and-mouth disease (HFMD) is one of the most common pediatric infectious diseases worldwide and it has affected up to two million children annually in China since 2008. This study used Bayesian spatiotemporal modeling to analyze provincial-level HFMD cases, environmental and socioeconomic data during 2009–2018. We found that both environmental (temperature, relative humidity and normalized difference vegetation index) and socioeconomic covariates (population density, birth rate, real GDP per capita and school vacation) are associated with HFMD occurrence under spatiotemporal scales. This model may help to (1) identify outbreaks of HFMD, including their timing, location and severity; (2) understand the transmission dynamics by mapping the spread of HFMD over time and space; (3) indicate areas of higher risk or the emergence of new outbreaks by detecting clusters of HFMD cases in specific geographic locations. Overall, this study provides valuable information for public health decision-makers and researchers, allowing them to better understand the spread of the disease and target their efforts more efficiently to control its transmission and prevent outbreaks.

## Introduction

Globally, hand-foot-and-mouth disease (HFMD) is one of the most common pediatric infectious diseases, and remains a major public health concern across the Asia-Pacific region pandemic potential [1]. In China, since its first large-scale epidemic in Fuyang City, Anhui Province in 2008 [2], HFMD has affected up to two million children annually [3,4]. Children younger than 5 years are especially prone to HFMD. Although most cases show mild and self-limiting illness, HFMD has the highest mortality rate among class ‘C’ infectious diseases in China, due to severe neurological and pulmonary complications [4]. HFMD is mainly caused by infection of human enterovirus (EV) A species. Since 2013 in China, coxsackievirus-A6 (CV-A6) and CV-A10 have become the predominant human EV serotypes over enterovirus A71 (EV-A71) and CV-A16 [5,6]. It was consistent with the worldwide (including Europe) outbreak of CV-A6 in 2013 [7]. EV-A71 vaccine has been available since 2016 and is expected to reduce EV71-related HFMD cases gradually. Low vaccine acceptance (< 50%) among the parents may be a hurdle for general EV71 vaccination among young children [8]. Moreover, for the full control of HFMD caused by several viral serotypes, development of multivalent vaccines is urgently needed [9].

Significant geographic disparities among China’s Eastern, Central, and Western regions pose challenges to disease control policies [10]. Previous studies suggested the spatial heterogeneity of HFMD and some possible influencing factors such as environmental and socioeconomic variables [11]. Some studies found that climate factors (i.e. temperature, relative humidity and wind speed) may be associated with HFMD transmission and socioeconomic factors (i.e. gross domestic product [GDP], ratio of urban to rural population and birth population) may be associated with HFMD transmission [12–16]. However, most studies were performed at a city scale or a provincial scale, and did not include analyses of socioeconomic factors. Moreover, spatiotemporal analyses at the national scale are scarce.

Given the high endemicity and dynamic complexity of HFMD, our study had three main objectives: (1) to detect and quantify the spatial heterogeneity of HFMD based on monthly data for 10 years from 31 provinces in China; (2) to construct the Bayesian spatiotemporal model to estimate the HFMD risk and evaluate model performance; and (3) to identify the driving factors on HFMD and quantify their effects in different regions.

## Materials and methods

### Data sources

Monthly data on HFMD cases from 31 provinces in China during 2009–2018 were collected from public health science data center website (www.phsciencedata.cn) of Chinese Center for Disease Control and Prevention (CCDC) [17]. The meteorological data were retrieved from China Surface Climatic Data Daily Data Set (V3.0) by China Meteorological Data Network (data.cma.cn/en), consisted of the observation data from 824 meteorological stations nationwide [18]. The socioeconomic data were obtained from the National Bureau of Statistics of China (www.stats.gov.cn) [19]. The normalized difference vegetation index (NDVI, range from -1 to 1) were retrieved from satellite images provided in the Moderate Resolution Imaging Spectroradiometer (MODIS) product MOD13Q1 (https://giovanni.gsfc.nasa.gov/)

### Temporal analyses

Seasonal analysis of HFMD incidence used X-12 Autoregressive Integrated Moving Average model (ARIMA), which was the most widely used method for decomposing time series into a trend, seasonal, and residual components [20]. Morlet wavelet analysis was performed to detect and quantify the periodicity of the time series of HFMD incidence [21]. Seasonal index was calculated as ratio of the average incidence rate to the average incidence rate per month. Heat maps of monthly HFMD incidence were created to identify the peak seasons in different provinces. A seasonal multiple linear regression model was adopted, using harmonic terms representing annual and semiannual periodic cycles, to estimate the peak timing and amplitude of the annual and semi-annual periodicities of HFMD activity in each province [3].

### Spatial analyses

The spatial heterogeneity of HFMD was judged of by plotting Lorenz curves and calculating the corresponding Gini indices [22]. The cumulative percentage of population and the cumulative percentage of HFMD cases were plotted graphically, reflecting the imbalance degree in the cases distribution. The Gini index (0–1) summarizes the statistics produced by the Lorentz curve. The value of Gini index close to 1 indicates a high degree of spatial heterogeneity.

The global Moran’s I index was used to measure global spatial autocorrelation based on both HFMD locations and incidence values simultaneously [23]. The value of Moran’s *I* index ranges from -1 to 1. If the index is significantly greater than 0, it indicates spatial agglomeration among provinces. A negative value indicates spatial difference.

We also perform the hot spot analysis, using ArcGIS Pro version 2.5 (ESRI Inc., Redlands, CA, USA), to identify local spatial autocorrelation [24]. Specifically, Getis-Ord Gi* statistic was calculated for local odds ratio (OR) maps (i.e., hot spot maps) of HFMD incidence in this study.

### Space-time scan analyses

The Kulldorff’s retrospective space-time scan statistic [25], based on the discrete Poisson probability model, was applied to detect the geographic areas and time periods of potential clusters with a significantly higher incidence of HFMD than those of neighboring areas. The shape of space-time scanning windows (clusters) is cylinder with radius (geographic) and height (time). The relative risk (RR) and log-likelihood ratio (LLR) were computed. The maximum radius of the circular base was set to 50% of the total population at risk and the maximum height of the cylinder was set to 50% of the total study period. The analyses were performed with SaTScan version 10.1 (Kulldorff and Information Management Services Inc, Cambridge, MA, USA). Then, ArcGIS was used to visualize the relative risk (RR) of HFMD in high-risk cluster areas.

### Spatiotemporal modeling and analyses

All the variables collected in this study were initially screened by the multicollinearity test using variance inflation factor (VIF) [26]. If VIF value of the variable > 10, it would be rejected. Bayesian model averaging (BMA) approach was applied for further variable selection [27]. It may identify the best-performing model, based on posterior probability as the weight to average all plausible models considered.

To explore the relationship of environmental, socioeconomic covariates and HFMD risk, we considered four types of regression models that responded to monthly HFMD cases for 31 provinces in China over 2009–18. Model 1 was the ordinary multiple linear regression (MLR) [28]. Model 2 was distributed lag nonlinear model (DLNM) [11]. Model 3 was the hierarchical Bayesian spatiotemporal model (HBSTM) adjusted for the temporal and spatial confounding effects [29]. We used a penalty complexity priors approach for all temporal and spatial random effects hyperparameters [30]. Model 4 was a combination of HBSTM and DLNM to making further interpretations of nonlinear effects of environmental covariates [31]. For each model, a negative binomial distribution was used to account for over-dispersion of HFMD case counts [26]. The goodness of fit (GoF) was assessed by the Watanabe-Akaike information criterion (WAIC), and the deviance information criterion (DIC), for which smaller values indicate better model fit [32]. The analyses were performed using R software version 4.2.1 (R Foundation for Statistical Computing, Vienna, Austria), mainly with the packages “bma”, “inla” and “dlnm”.

### Cross-validation

An essential step in establishing realistic epidemic models is cross-validating them against historical spread of outbreaks and underlying patterns [33]. The out-of-sample predicted HFMD incidence rates from Jan 2009 to Dec 2018, as the posterior means and upper 95% credible intervals, were calculated from the final HBSTM+DLNM model (fitted 10 × 12 months, excluding the month for which the prediction is valid)). We estimated the moving outbreak thresholds as the 75^th^ percentile of the distribution of HFMD cases per month over the 120-month period, excluding 1 month at a time. The moving thresholds were applied to produce predicted probabilities of exceeding the outbreak threshold for all 10 years of 2009–2018.

## Results

### Temporal, spatial and population distribution of HFMD

**Fig 1:**
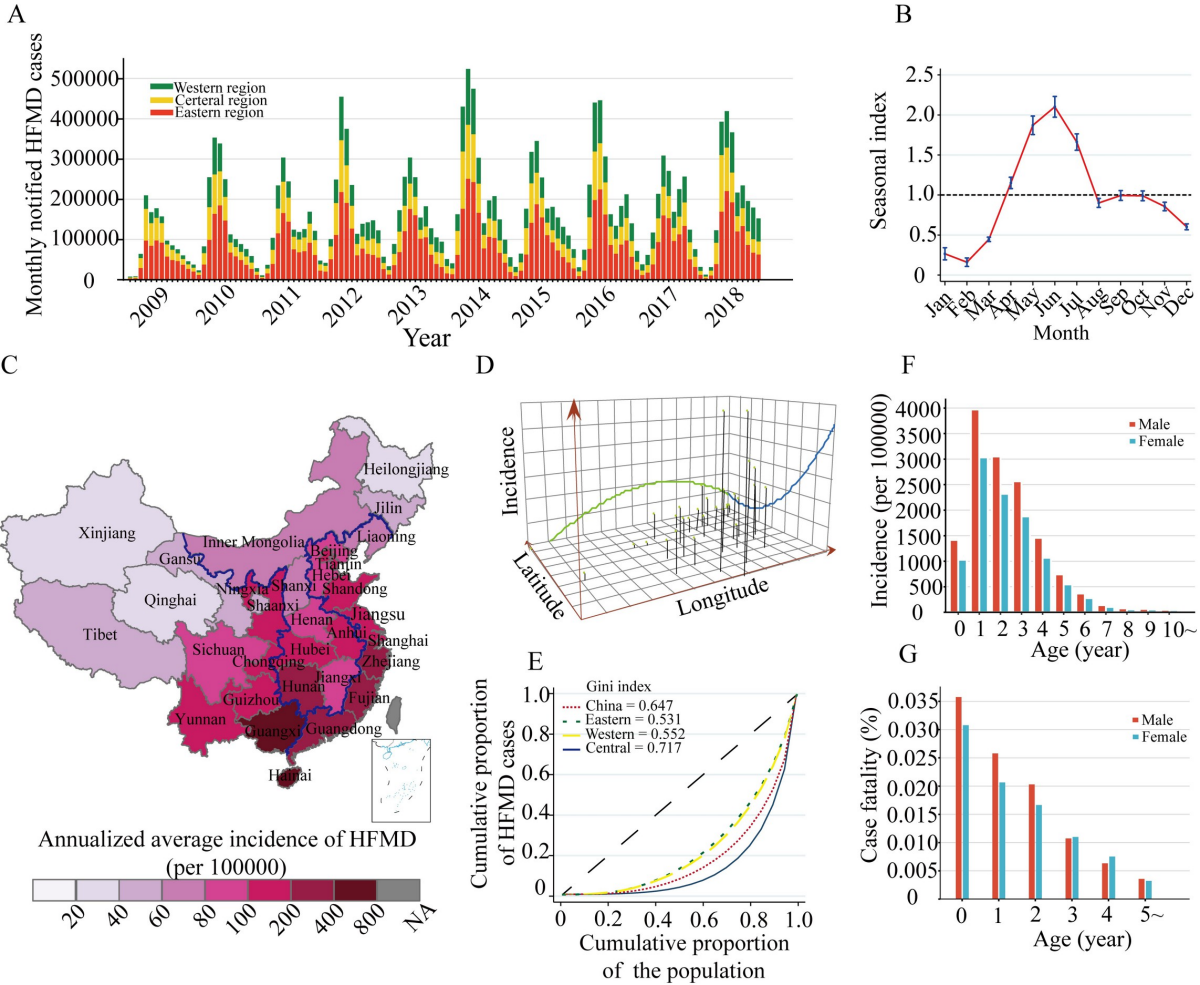
Temporal, spatial and population distribution of HFMD in China during 2009–2018. (A) Time series of monthly HFMD incidence. (B) The seasonality index is calculated as the ratio of the average number of cases in a given month to the average number of cases per month over 10×12 months, and its 95% CI is calculated using SD. A seasonality index greater than 1.0 indicates a seasonal trend. (C) The geographic distribution of HFMD incidence. The Western region includes 11 provinces located in the interior of China: Shaanxi, Gansu, Qinghai, Ningxia, Xinjiang, Sichuan, Chongqing, Yunnan, Guizhou, Guangxi and Tibet. The Central region includes 9 provinces: Shanxi, Jilin, Heilongjiang, Inner Mongolia, Anhui, Jiangxi, Henai, Hubei, and Hunan. The Eastern region includes 11 coastal provinces: Liaoning, Hebei, Beijing, Tianjin, Shangdong, Jiangsu, Shanghai, Zhejiang, Fujian, Guangdong, and Hainan. The image is generated in ArcGIS Pro version 2.5 (ESRI Inc., Redlands, CA, USA), using a freely downloaded shapefile from the National Geomatics Center of China (https://www.ngcc.cn/ngcc/html/1/). (D) Three-dimensional trend of the average annual HFMD incidence. (E) Lorenz curves of the distribution HFMD cases as a function of population size at the province level. The black line (first diagonal) represents a constant distribution of HFMD cases (no heterogeneity). Age-gender distribution of reported HFMD cases (F) and case fatality rate (G).

Throughout 2009–2018, a total of 20048244 HFMD cases were reported from 31 provinces in mainland China (Table A in S1 File and Fig 1A). The seasonal index of 12 months ranged from 0.16 to 2.09 and the annual epidemic peak was observed from April to July (Fig 1B). The time-series seasonal decomposition analysis showed a significant seasonal periodicity (Fig A-A in S1 File). The wavelet spectrum power analysis at the national level revealed a pronounced band at 1 y and an inconsistent band at 0.5 y, suggesting that the long-term temporal variation of HFMD is dominated by the annual pattern (Fig A-B in S1 File). When the data are stratified by geographic region, the Western, Central and Eastern regions represented low, moderate, and high level of transmission intensity (Fig 1C). The three-dimensional trend of the average annual incidence showed an ascending arc trend from north to south (Fig 1D). The Lorenz curves showed that Central region had a higher degree of spatial heterogeneity larger than Eastern and Western regions (Fig 1E). Children under 5 years of age had the highest average incidence of HFMD (22.3 cases per 1000 population), while the boys had 1.35 times higher incidence than the girls (25.4 vs 18.8 cases per 1000 population, *P* < 0.001) (Fig 1F). The case-fatality rate of HFMD (overall 0.019%) was highest among children aged < 12 months (0.034%) (Fig 1G).

### Regional HFMD seasonal patterns

**Fig 2:**
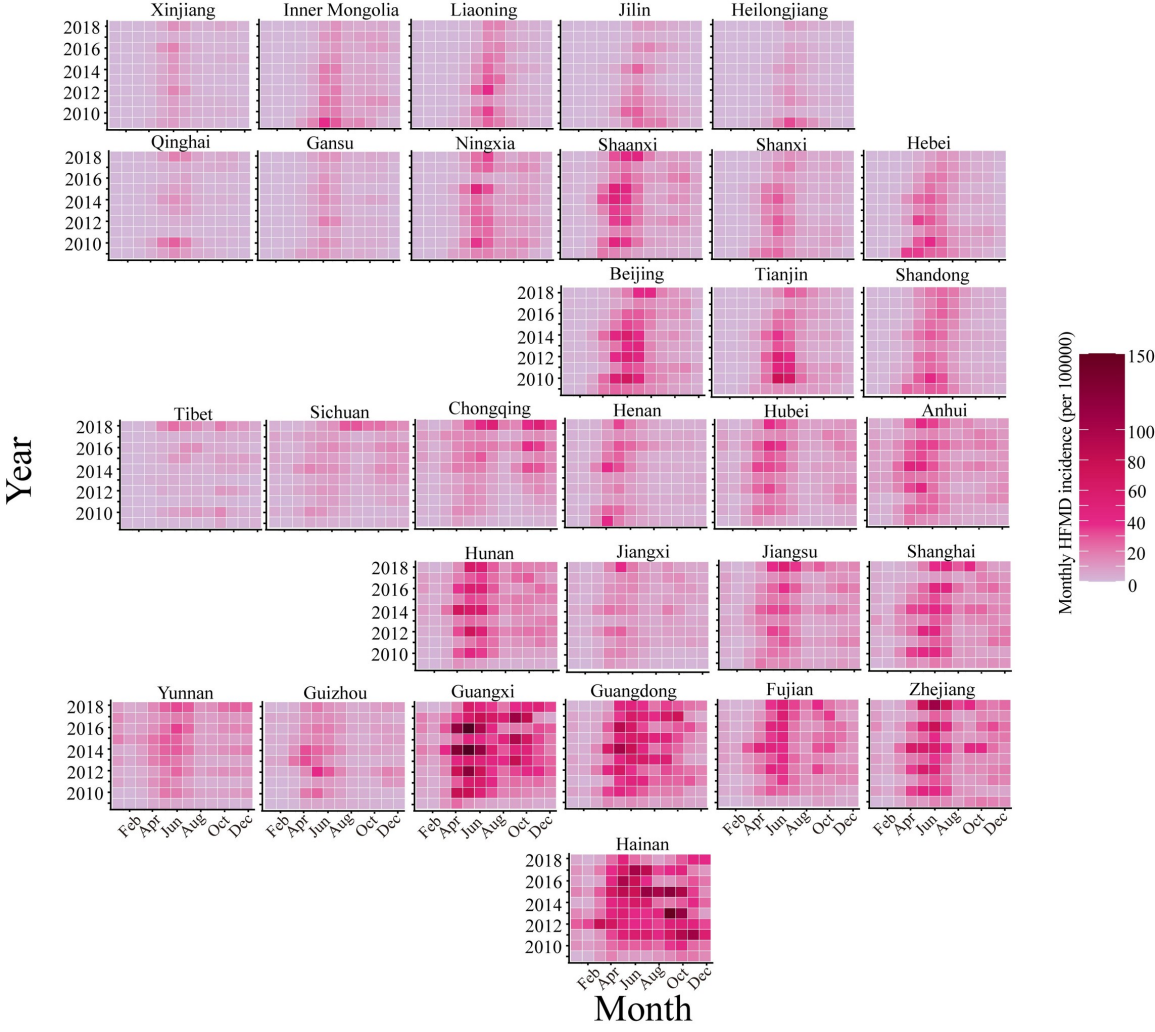
Spatial and temporal variation in HFMD incidence in China during 2009–2018. Monthly HFMD incidence rates (per 100000 people) between January, 2009, and December, 2018 were aggregated at the province level. Provinces were ordered by their geographical location.

**Fig 3:**
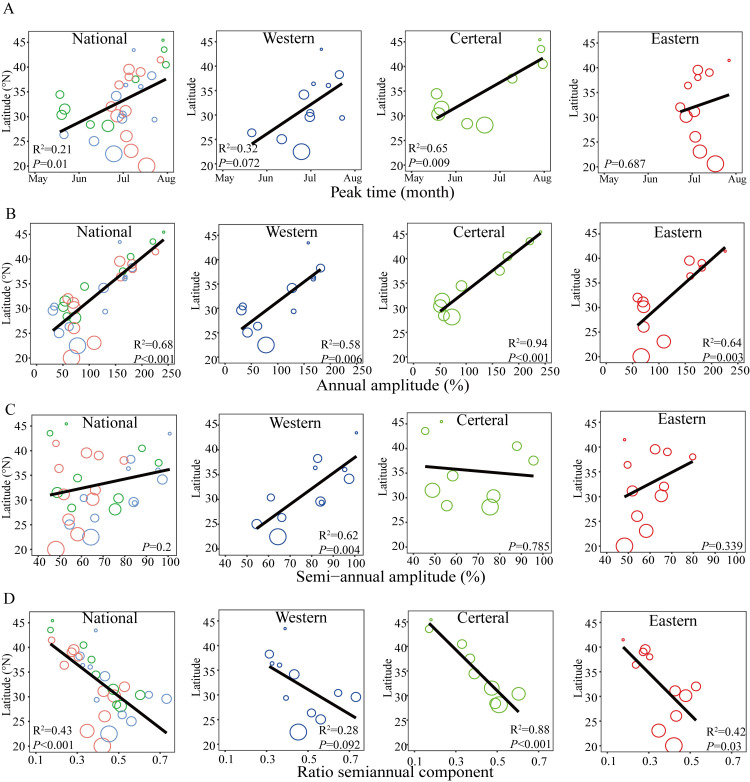
Latitudinal gradients in seasonality of HFMD in China. (A) Peak time. (B) Annual amplitude. (C) Semiannual amplitude. (D) Ratio semiannual component (higher ratio suggests a stronger semiannual periodicity). Symbol size is proportional to the number of cases in each province. Black solid lines represent linear regression fit. Colors represent different geographic regions (red = Eastern region, green = Central region, blue = Western region).

Over the 10-year period, HFMD incidence has remained relatively steady. HFMD seasonality varies across the country, with the peak transmission season occurring earlier in the year in the south (Fig 2). We modeled the seasonal models based on negative binomial (NB) generalized linear models (GLMs) with harmonic terms [34]. The median model fitness (R^2^) for all provinces reached 0.63 (range 0.28–0.81) (Fig B in S1 File). The estimating seasonality displayed significant latitudinal gradients in peak time, annual amplitude, and contribution of semi-annual periodicity (measured by the ratio of the semi-annual amplitude to the sum of the annual and semi-annual amplitudes) of HFMD in China. The gradients in Central region were stronger than those in the other two regions, suggesting higher geographic dependence (*P* < 0.05, (Figs 3 and C in S1 File).

### Spatial and spatiotemporal clustering of HFMD

We identified significant positive spatial autocorrelation for HFMD incidence at the provincial level during 2009–2018. Global Moran’s I index ranged from 0.416 to 0.608 (Table B in S1 File), indicating that HFMD incidence was geographically clustered. Based on local indicators of spatial autocorrelation analysis, the hot spots (high-high cluster) were observed in the north in 2009, covering seven provinces. Changes were observed since 2010 when the high-risk cluster moved toward south, and since 2014 when the cold spots (low-low cluster) were moving eastward (Fig D in S1 File).

**Fig 4:**
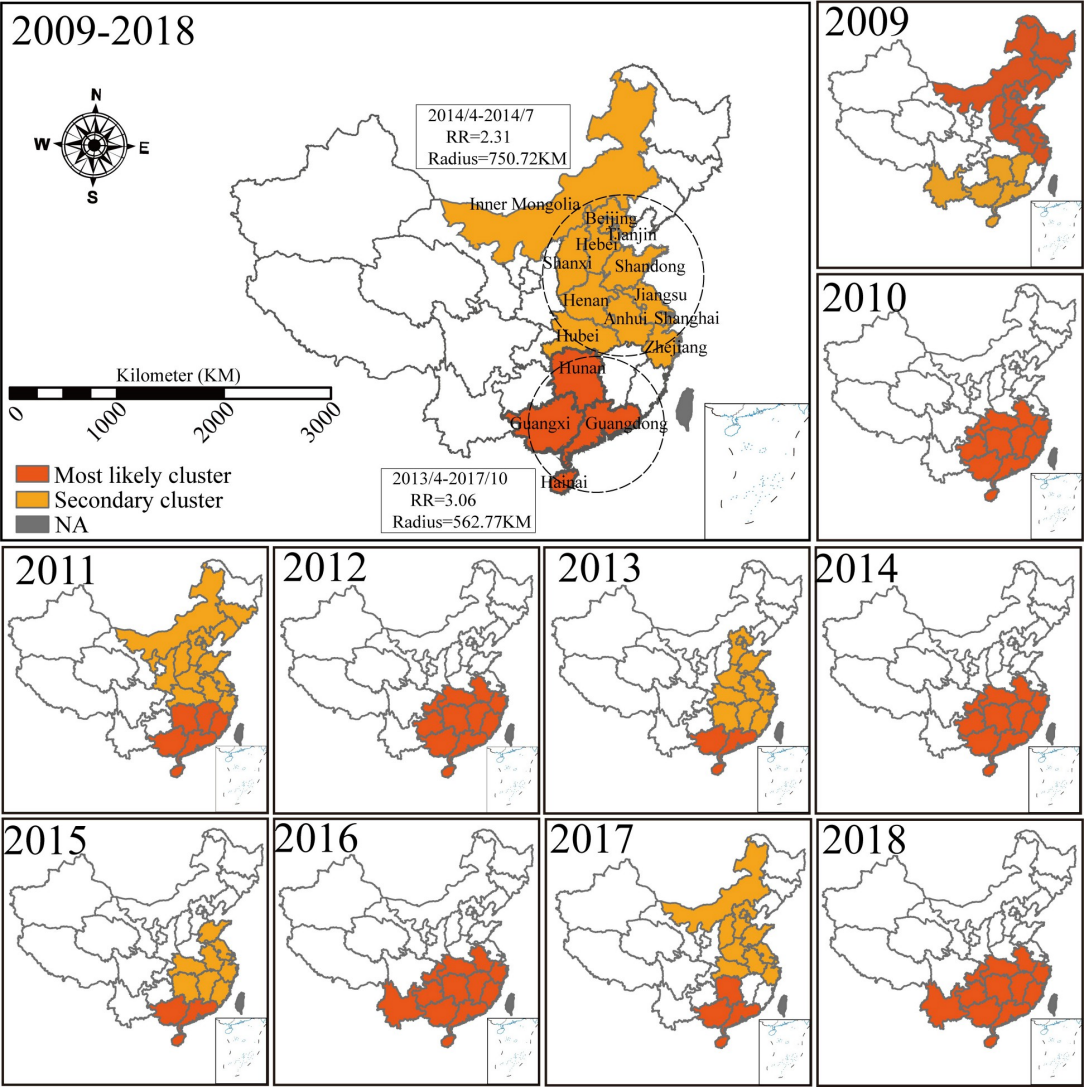
Spatiotemporal clustering of HFMD incidents in China from 2009 to 2018. Kulldorff’s space-time scan statistic was used to identify the geographic areas and time periods of potential clusters with significantly higher incidence of HFMD than those of neighboring areas. The window with the maximum log-likelihood ratio (LLR) was defined as the most likely cluster area, and other windows with significant LLR as the secondary potential clusters. A statistically significant assessment of 9999 replicates was performed using Monte Carlo simulation with a significance level of 0.05. For other parameters, the maximum radius of the base of the circle is set to 50% of the total population at risk and the maximum height of the cylinder is set to 50% of the total study period. More detailed information is provided in Table C in S1 File. The image is generated in Arcgis Pro version 2.5 (ESRI Inc., Redlands, CA, USA), using a freely downloaded shapefile from the National Geomatics Center of China (https://www.ngcc.cn/ngcc/html/1/).

Based on Kulldorff’s space-time scan analysis [35], one most likely high-risk spatiotemporal clustering area and 1–2 secondary-risk clustering areas were detected annually in China during 2009–2018. The most likely cluster areas for HFMD were located in south China (Guangdong, Guangxi, Hunan, Hainan) from April 2013 to October 2017. The secondary cluster areas were located in some areas of central and east China (Beijing, Tianjin, Hebei, Shandong, Jiangsu, Shanghai, Zhejiang) from April to July 2014 (Fig 4 and Table C in S1 File).

### Model selection and evaluation

Using variance inflation factor (VIF) as a screening tool, four covariates with VIF > 10 were considered to be collinear and were removed (Fig E and Table D in S1 File). Further, the Bayesian model averaging (BMA) approach was applied to the regression models. As shown in Table E in S1 File, compared with other selected models, model 1 (considering nine explanatory variables) had the highest posterior model probability (PMP) (50.0% of the total posterior probability).

Moving from traditional linear and nonlinear model (MLR and DLNM), we noted progressive improvements in model fitting and predictive performance for hierarchical Bayesian spatiotemporal model and its incorporation form with DLNM (Table 1). To select the best fitting models, we included province-level monthly random effects, to account for varying seasonality between different areas (Fig F in S1 File), and year-specific spatial random effects to account for unexplained interannual spatial heterogeneity (Fig G in S1 File).

**Table 1:** Bayesian modeling evaluations of the alternative regressions for China’s HFMD case account for model fitness complexity and predictive power.

	Main Method	DIC	WAIC	*P*_DIC_	*P*_WAIC_	RMSE	R^2^
Model 1	MLR	64969.64	64971.52	11.92	13.67	11.221	0.562
Model 2	DLNM	63916.63	63929.81	49.19	60.12	10.910	0.598
Model 3	HBSTM	60888.05	60921.74	593.35	542.08	6.629	0.856
Model 4	HBSTM+DLNM	60762.65	60817.18	612.53	572.99	6.484	0.869

MLR multiple linear regression; DLNM distributed lag nonlinear model. HBSTM hierarchical Bayesian spatiotemporal model

DIC (deviance information criterion) and WAIC (Watanabe-Akaike information criterion) were used for reflecting the degree of model fitting; P_DIC_ and P_WAIC_ (effective number of parameters from DIC and AIC) represented complexity of regression and model performance; RMSE (root mean square error) and R^2^ (Squared correlation coefficient) retrieved from the conditional predictive ordinates under a leave-one-out cross-validation was reflective of the predictive power.

### Detection of outbreaks

**Fig 5:**
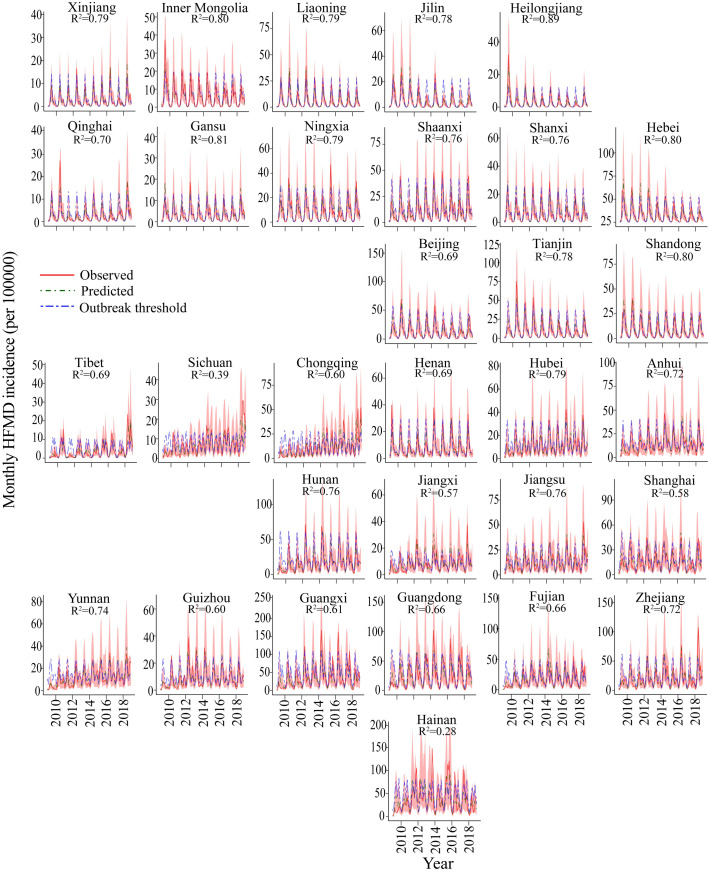
Predicted versus observed HFMD incidence rates per province. Mean observed HFMD incidence rate per 100000 people (black curve), and corresponding mean out-of-sample predicted incidence rate (red curve) and 95% credible interval (shaded area) simulated from the HBSTM+DLNM model (refitted 12 × 10 months from Jan 2009 to Dec 2018, excluding the month for which the prediction was valid). Moving outbreak thresholds (blue curve) were calculated as the 75^th^ percentile of the distribution of HFMD cases per month over the 120-month period, leaving out one month at a time. Provinces are ordered by their geographical location.

**Fig 6:**
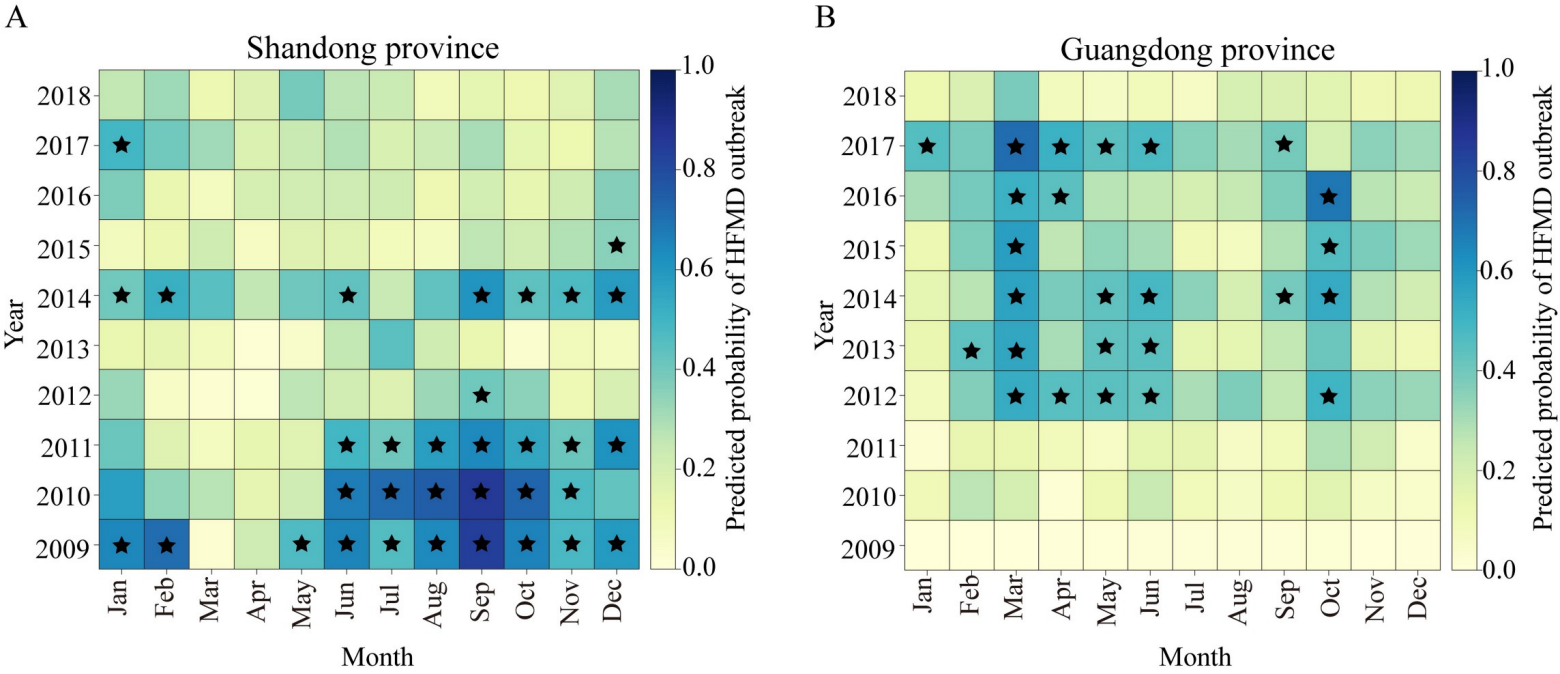
Predicted probability of exceeding moving outbreak thresholds. Predicted probability of observing a HFMD outbreak in Shandong(A) and Guangdong (B) provinces in China. The moving outbreak thresholds were calculated as the 75^th^ percentile of observed HFMD cases per month from Jan 2009 to Dec 2018, excluding the month for which the prediction is valid. The out-of-sample predictive distributions were simulated from the HBSTM+DLNM model. The graduated color bars represent the predicted probabilities ranging from 0 (pale colors) to 1 (deep colors). The months in which the moving outbreak threshold was exceeded are marked with a pentagram.

The models explicitly incorporated data on main environmental and socioeconomic covariates, and showed the out-of-sample predicted vs. observed HFMD incidence rates (per 100000 population) at the provincial level for China. We found that our models could correctly capture the general timing and intensity of HFMD outbreaks, except in two provinces of Sichuan and Hainan (median goodness-of-fit R^2^ = 0.78, range 0.28–0.89, Fig 5). Fig 6 shows the probability of exceeding the moving outbreak threshold in two representative provinces (i.e., Shandong and Guangdong). It well distinguished between outbreak and non-outbreak months throughout the ten years.

### Combined impacts of environmental and socioeconomic covariates

Two kinds of overall impacts of environmental and socioeconomic covariates on HFMD dynamic were estimated. One was the linear effects based on HBSTM and the other was nonlinear and delayed effects obtained from HBSTM+DLNM. We summarized the critical parameters of the linear HBSTM and HBSTM+DLNM in Table 2, including the overall coefficients and their relative risks. This HBSTM explained 85.6% of the variance in HFMD transmission during 2009–2018 while HBSTM+DLNM accounted for 86.9%. In terms of the environmental covariates, temperature, relative humidity and normalized difference vegetation index (NDVI) displayed as significant positive stimulants for HFMD transmission. Besides, among the socioeconomic covariates, the population density, birth rate, real GDP per capita demonstrated positive association with HFMD incidence while school vacation served as a restraining force (nearly half reduction).

**Table 2:** Linear impacts of environmental and socioeconomic covariates on HFMD risk in China during 2009–2018.

Variables	Identifier	HBSTM		HBSTM+DLNM	
		Coeff, poster mean (95%CI)	RR (95%CI)	Coeff, poster mean (95%CI)	RR (95%CI)
α (intercept)		**-1.586 (-2.716,-0.448)**	/	**-1.784 (-2.957,-0.608)**	/
Temperature	EC1	**0.052 (0.042,0.062)**	**1.053 (1.043,1.064)**	/	see Fig 4
Relative humidity	EC2	**0.015 (0.011,0.018)**	**1.015 (1.011,1.018)**	/	
Wind speed	EC3	0.027 (-0.054,0.107)	1.027 (0.948,1.113)	/	
NDVI	EC8	**1.317 (0.857,1.778)**	**3.732 (2.356,5.919)**	/	
Log (population density)	SC1	**0.127 (0.052,0.201)**	**1.136 (1.054,1.222)**	**0.232 (0.156,0.307)**	**1.261 (1.169,1.353)**
Birth rate	SC2	**0.051 (0.021,0.081)**	**1.052 (1.022,1.084)**	**0.056 (0.025,0.086)**	**1.058 (1.025,1.090)**
Log (real GDP per capita)	SC5	**0.140 (0.027,0.253)**	**1.150 (1.027,1.288)**	**0.151 (0.032,0.270)**	**1.163 (1.033,1.310)**
Log (passenger volume)	SC7	**-0.112 (-0.179, -0.045)**	**0.894 (0.836,0.956)**	-0.031 (-0.102,0.040)	0.969 (0.903,1.041)
Beds of medical institutions	SC9	-0.002 (-0.007,0.004)	0.998 (0.993,1.003)	-0.003 (-0.009,0.002)	0.997 (0.991,1.002)
School vacation	SC10	**-0.646 (-0.748, -0.543)**	**0.524 (0.473,0.581)**	**-0.680 (-0.779,-0.582)**	**0.507 (0.459,0.559)**
Total explained variance (R^2^)		0.856 (*P*<0.001)		0.869 (*P*<0.001)	

Estimates in bold format indicate statistical significant results with *P* value < 0.05.

HBSTM, hierarchical Bayesian spatiotemporal model; DLNM, distributed lag nonlinear model; RR, relative risk; NDVI, normalized difference vegetation index.

**Fig 7:**
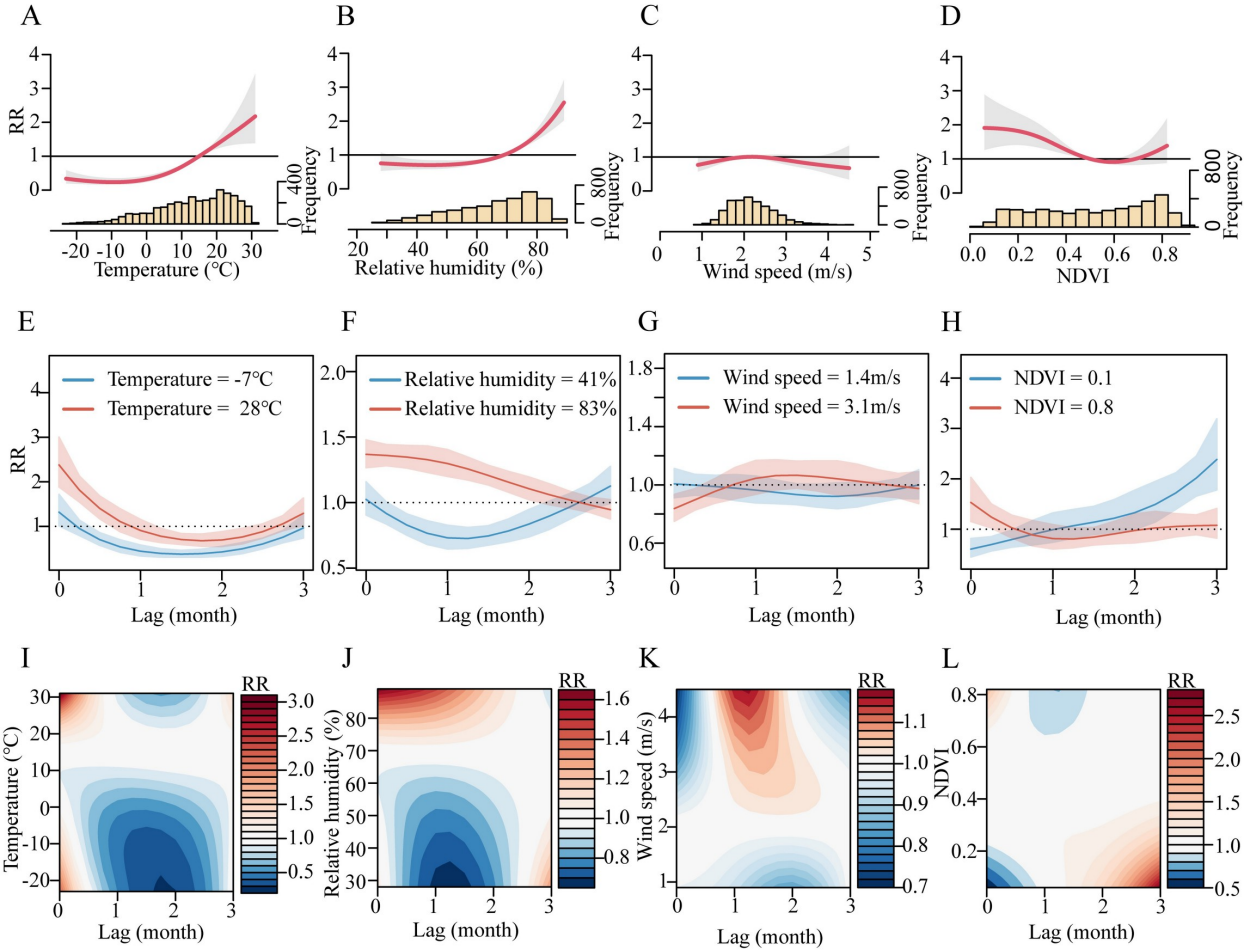
Nonlinear and delayed impacts of environmental covariates on HFMD risk. (A-D) Cumulative risks over the 3-month period. Lines and shaded areas showed the relative risk (RR) and their 95%CI for the association between each environmental covariate and HFMD incidence, relative to that covariate’s median. (E-H) Lag-response association for the extreme values (5% and 95% percentiles) of the environmental covariates. (I-L) Contour plots of the exposure-lag-response association between the environmental covariates and HFMD incidence.

Furthermore, we found significant nonlinear associations of environmental covariates and HFMD transmission, allowing interaction with underlying socioeconomic conditions (Fig 7). The results of the linear model (Table 2) seemed to obscure the nonlinear trends of such associations. The nonlinear trends varied by covariates. Fig 7A and 7B captured the thresholds of the climate covariates (monthly average temperature of 2°C, monthly average relative humidity of 60%) when their effects started to change markedly. In the linear regression model, higher NDVI was associated with higher risk of HFMD; however the direction of this association reversed in the nonlinear model. Fig 7D shows a negative association between NDVI and HFMD risk and the curve became relatively flat for the value range beyond 0.6. Fig 7E–7H shows the lag-response association for the extreme values (5% and 95% percentiles) of the environmental covariates. Fig 7I–7L were contour plots of the exposure-lag-response association between the environmental covariates and HFMD risk. In this context, population density (RR = 1.261; 95%CI, 1.169–1.353), birth rate (RR = 1.058; 95%CI, 1.025–1.090), real GDP per capita (RR = 1.163; 95%CI, 1.033–1.310) and school vacation (RR = 0.507; 95%CI, 0.459–0.559) were identified to have positive or negative effects on HFMD respectively (Table 2). Finally, we obtained a provincial estimate of the incidence of Chinese mainland HFMD in the 10-year period 2009–2018 through this model. The darker the province, the higher the incidence. The incidence predicted by the model was close to the actual incidence result, and the incidence center gradually shifted from the north to the south (Fig H in S1 File).

In summary, our study expanded the limited knowledge of the associations between various covariates and HFMD in China from a spatiotemporal heterogeneous perspective. It may prove valuable for advancing our understanding of complex HFMD transmission system, improving decision-making and addressing global challenges. The framework could provide implications for research on other disease-environment systems.

## Discussion

There are rare studies on the spatiotemporal patterns of HFMD transmission at a national scale, but that could prove valuable in the design of geographically-tailored surveillance and intervention strategies such as vaccination. We used HFMD incidence data of China, a geographically diverse country, to quantify spatiotemporal patterns of incidence and clustering for HFMD during the period 2009–2018. It is not surprising that our results support a diversity of HFMD epidemic patterns which are driven by both environmental and socioeconomic covariates. In this study, we developed a statistical model which could successfully predict months with HFMD outbreaks versus non-outbreaks in provinces of China from Jan 2009 to Dec 2018. In practice, the CCDC may use this model to generate disease forecasts of HFMD using the weather forecast products and socioeconomic parameters. Public health decision-makers could use such forecasts as an early warning tool to plan interventions to reduce risk of HFMD and other infectious diseases.

We quantified spatial heterogeneity across geographic regions using Lorenz curves and their corresponding Gini indices. The underlying distributions of HFMD seasonal peaks and epidemic amplitudes across China are also characterized. The spatial heterogeneity was highest in the Central regions. Meanwhile, HFMD had the highest latitude-dependency of the seasonal trends and characteristics within the same region. Such results highlighted the importance of incorporating geographic dimension in appropriately disentangling and interpreting the relevant seasonal patterns and distribution of infectious diseases. That is, data aggregation may complicate our understanding of the HFMD dynamics in China. The complication may be resulted from epidemiological patterns which differ across spatially heterogeneous areas.

Earlier studies suggested simple linear relationships between temperature, relative humidity and HFMD incidence in Japan [36], Hong Kong [37], Shandong province [38] and Guangzhou city [39], China. Other studies showed that HFMD was associated with lagged climate covariates in Beijing and Changsha of China [40,41]. Consistent with these studies, we found that hot and humid climate may be a preferred environment for HFMD transmission. Possible explanation relies on the virological evidence that both temperature and humidity are crucial for the survival of EV [42]. Low temperature (< 2°C) may not favor the survival of virus and personal close contacts, which may hinder the transmission of HFMD. Higher humidity allows the virus to persist longer on inanimate surfaces [43]. Furthermore, in the temporal dimension, the disease-climate associations between HFMD and temperature, relative humidity were very similar to the temporal trend of HFMD itself, indicating that the two climate covariates be better explanatory factors for HFMD incidence at a localized temporal scale.

Studies on association between the NDVI and HFMD risk are scarce. Two studies reported a negative association, with each 1σ increase in NDVI reducing the incidence of HFMD by 20% and 37% [44,45], while another one suggested a positive association [46]. We found a negative nonlinear relationship between the two. Besides, it is worth noting that the NDVI increased in the spring and summer, coincided with the HFMD season, thus reflecting a positive association (in the linear model). Our study advanced the previous studies by using hierarchical Bayesian models to assess the delayed and nonlinear effects of NDVI. A study found that high NDVI may be associated with enhanced physical, psychological, and social well-being that can improve immunity, such as by providing a visually complex environment that reduces stress and mental fatigue, or by adding the look and feel of a place to provide a pleasant location for social or physical activity [47].

In regards to socioeconomic conditions, most covariates we tested were positively associated with the HFMD incidence in China, i.e., the population density, birth rate and per capita GDP. In developed areas, the higher population density leads to an easy spread of the virus. The school vacation was the only socioeconomic covariate that was markedly negatively correlated with HFMD incidence, possibly because the reduced the close contact of children [48]. Another study found that school holidays did not substantially reduced HFMD transmission in Hong Kong [49]. This might be explained by the fact that Hong Kong has a number of breaks and school holidays which vary every year, different from those in mainland China. The population-level findings in this study suggest that some measures such as the annual school vacation may be critical to reducing the incidence of HFMD.

For effective disease forecasting models, it is vital to identify the key drivers, lag periods, and appropriate model formulation that reflects the local disease transmission ecology. In addition, it takes time for anomalies in the covariates to manifest and contribute to disease risk. Our study selected a model to infer combined impacts of environmental and socioeconomic covariates on HFMD risk and predict the probability of exceeding an outbreak threshold. The translation of probabilities into discrete warnings needs to be carried out with caution and reflecting the capacity of the public health system to respond to imminent outbreaks.

Admittedly, there are several limitations which should be better addressed in future research. First, due to a lack of available data, there might be some important effect modifiers that we failed to take into consideration, such as viral type, reproduction number and public health interventions. For example, the inactivated EV-A71 vaccine has shown certain efficacy in reducing the incidence of EV71-related HFMD among the vaccination population [50]. Due to changes in dominant disease-causing virus types [5] as well as the short application (since 2016) and low vaccination rate [8], the vaccination program seemed not to cause substantial changes in HFMD epidemics and thus were not considered in the current analysis. Second, our analysis quantified the associations between the influential covariates and HFMD transmission, but such associations were not necessarily causal. For example, the GDP per capita may just represent economic activities, which were irrelevant to transmission. Third, HFMD cases and covariates may vary across different cities within the province. City-level comparative studies are warranted to address this issue which is particularly important for a vast province with unbalanced development. Last but not least, the relative risk map for the future trends of HFMD has not been projected. Actually, in the context of ongoing COVID-19 pandemic, the behaviors of many infectious diseases have been changed [51,52].

In summary, our study expanded the limited knowledge of the associations between various covariates and HFMD in China from a spatiotemporal heterogeneous perspective. It may prove valuable for advancing our understanding of complex HFMD transmission system, improving decision-making and addressing global challenges. The framework could provide implications for research on other disease-environment systems.

## Supporting information

S1 File Table A in S1 File: Characteristics of all HFMD cases in China from 2009 to 2018. Fig A in S1 File: Seasonality and periodicity of HFMD cases in China during 2009–2018. Fig B in S1 File: Fit of seasonal models in 31 provinces in China. Fig C in S1 File: Seasonal estimates of HFMD in China during 2009–2018. Table B in S1 File: Global spatial autocorrelation analysis. Fig D in S1 File: Local hotspot clusters of the annual incidence of HFMD in China during 2009–2018. Table C in S1 File: Spatiotemporal clustering of HFMD incidents in China during 2009–2018. Table D in S1 File: Province-level potential explanatory variables of regional HFMD in China: EC1-EC8 denote environmental factors and SC1-SC10 denote socioeconomic factors. Fig E in S1 File: Variables screening procedure: remove variables with higher multicollinearity (VIF > 10). Table E in S1 File: BMA model selection. Fig F in S1 File: Contribution of the province-specific monthly random effects to HFMD incidence rate (IR) estimates. Fig G in S1 File: Contribution of year-specific spatial random effects to HFMD incidence rate (IR) estimates. Fig H in S1 File: Posterior predictive mean HFMD incidence rate 2009–2018.
(DOCX)
