# Supplementary material for: Combined impacts of environmental and socioeconomic covariates on HFMD risk in China: A spatiotemporal heterogeneous perspective
Source: PLoS Negl Trop Dis. 2023 May 19;17(5):e0011286. doi: 10.1371/journal.pntd.0011286 (PMC10198510; doi:10.1371/journal.pntd.0011286)
Supplement: S1 File — Table A in S1 File: Characteristics of all HFMD cases in China from 2009 to 2018. Fig A in S1 File: Seasonality and periodicity of HFMD cases in China during 2009–2018. Fig B in S1 File: Fit of seasonal models in 31 provinces in China. Fig C in S1 File: Seasonal estimates of HFMD in China during 2009–2018. Table B in S1 File: Global spatial autocorrelation analysis. Fig D in S1 File: Local hotspot clusters of the annual incidence of HFMD in China during 2009–2018. Table C in S1 File: Spatiotemporal clustering of HFMD incidents in China during 2009–2018. Table D in S1 File: Province-level potential explanatory variables of regional HFMD in China: EC1-EC8 denote environmental factors and SC1-SC10 denote socioeconomic factors. Fig E in S1 File: Variables screening procedure: remove variables with higher multicollinearity (VIF > 10). Table E in S1 File: BMA model selection. Fig F in S1 File: Contribution of the province-specific monthly random effects to HFMD incidence rate (IR) estimates. Fig G in S1 File: Contribution of year-specific spatial random effects to HFMD incidence rate (IR) estimates. Fig H in S1 File: Posterior predictive mean HFMD incidence rate 2009–2018. (DOCX) [file pntd.0011286.s001.docx]

**Supporting information S1_File:**

**Combined impacts of environmental and socioeconomic covariates on HFMD risk in China: A spatiotemporal heterogeneous perspective**

Chun-Hu Li,^1☯^ Jun-Jie Mao,^1☯^ You-Jia Wu,^2☯^ Bin Zhang,^3^ Xun Zhuang,^4^ Gang Qin,^1,3*^ Hong-Mei Liu^5*^

^1^Joint Division of Clinical Epidemiology, Affiliated Hospital of Nantong University, School of Public Health of Nantong University, Nantong, China

^2^Department of Pediatrics, Affiliated Hospital of Nantong University, Nantong, China

^3^Department of Infectious Diseases, Affiliated Hospital of Nantong University, Nantong, China

^4^Department of Epidemiology and Biostatistics, School of Public Health of Nantong University, Nantong, China

^5^School of Transportation and Civil Engineering of Nantong University, Nantong, China

^☯^Li, Mao and Wu contributed equally to this work.

* [tonygqin@ntu.edu.cn](mailto:tonygqin@ntu.edu.cn); [liu.hm@ntu.edu.cn](mailto:liu.hm@ntu.edu.cn)

Table of Contents

[**1. Temporal analyses** 3](#_Toc134624489)

[Table A. Characteristics of all HFMD cases in China from 2009 to 2018. 3](#_Toc134624490)

[Fig A. Seasonality and periodicity of HFMD cases in China during 2009-2018. 4](#_Toc134624491)

[Fig B. Fit of seasonal models in 31 provinces in China. 5](#_Toc134624492)

[Fig C. Seasonal estimates of HFMD in China during 2009-2018. 6](#_Toc134624493)

[**2. Spatial analyses** 7](#_Toc134624494)

[Table B. Global spatial autocorrelation analysis. 7](#_Toc134624495)

[Fig D. Local hotspot clusters of the annual incidence of HFMD in China during 2009-2018. 8](#_Toc134624496)

[**3. Space-time scan analyses** 9](#_Toc134624497)

[Table C. Spatiotemporal clustering of HFMD incidents in China during 2009-2018. 9](#_Toc134624498)

[**4. Spatiotemporal modeling and analyses** 10](#_Toc134624499)

[Table D. Province-level potential explanatory variables of regional HFMD in China: EC1-EC8 denote environmental factors and SC1-SC10 denote socioeconomic factors. 10](#_Toc134624500)

[Fig E. Variables screening procedure: remove variables with higher multicollinearity (VIF > 10). 11](#_Toc134624501)

[Table E. BMA model selection. 12](#_Toc134624502)

[Fig F. Contribution of the province-specific monthly random effects to HFMD incidence rate (IR) estimates. 13](#_Toc134624503)

[Fig G. Contribution of year-specific spatial random effects to HFMD incidence rate (IR) estimates. 14](#_Toc134624504)

[Fig H. Posterior predictive mean HFMD incidence rate 2009-2018. 15](#_Toc134624505)

# Temporal analyses

# Table A. Characteristics of all HFMD cases in China from 2009 to 2018.

|  | **Male** | **Female** | **Total** |
| --- | --- | --- | --- |
| Total cases, number (%) | 12228636(61.00) | 7819607(39.00) | 20048243 |
| Age groups, number of cases (%) |  |  |  |
| 0~ | 1185687(61.69) | 736301(38.31) | 1921988 |
| 1~ | 3717398(60.63) | 2414036(39.37) | 6131434 |
| 2~ | 2755143(61.03) | 1758953(38.97) | 4514096 |
| 3~ | 2136893(61.52) | 1336697(38.48) | 3473590 |
| 4~ | 1174560(61.64) | 730847(38.36) | 1905407 |
| 5~ | 571999(61.25) | 361885(38.75) | 933884 |
| 6~ | 270982(60.57) | 176378(39.43) | 447360 |
| 7~ | 123156(59.37) | 84289(40.63) | 207445 |
| 8~ | 76449(58.58) | 54053(41.42) | 130502 |
| 9~ | 52941(58.70) | 37255(41.30) | 90196 |
| 10~ | 163428(55.90) | 128913(44.10) | 292341 |
| Year, number of cases (%) |  |  |  |
| 2009 | 726235(62.85) | 429290(37.15) | 1155525 |
| 2010 | 1113080(62.72) | 661587(37.28) | 1774667 |
| 2011 | 1019937(62.97) | 599769(37.03) | 1619706 |
| 2012 | 1357441(62.59) | 811295(37.41) | 2168736 |
| 2013 | 1118941(61.20) | 709436(38.80) | 1828377 |
| 2014 | 1672498(60.19) | 1106363(39.81) | 2778861 |
| 2015 | 1206005(60.38) | 791366(39.62) | 1997371 |
| 2016 | 1462922(59.90) | 979216(40.10) | 2442138 |
| 2017 | 1151027(59.65) | 778523(40.35) | 1929550 |
| 2018 | 1400550(59.51) | 952760(40.49) | 2353310 |
| Age group, number of deaths (%) |  |  |  |
| 0~ | 424(65.13) | 227(34.87) | 651 |
| 1~ | 959(65.78) | 499(34.22) | 1458 |
| 2~ | 559(65.61) | 293(34.39) | 852 |
| 3~ | 231(61.11) | 147(38.89) | 378 |
| 4~ | 75(57.69) | 55(42.31) | 130 |
| 5~ | 45(62.50) | 27(37.50) | 72 |
| Season, number of cases (%) |  |  |  |
| Spring |  |  | 6190533(30.88) |
| Summer |  |  | 7595530(37.89) |
| Fall |  |  | 4507832(22.49) |
| Winter |  |  | 1753106(8.74) |


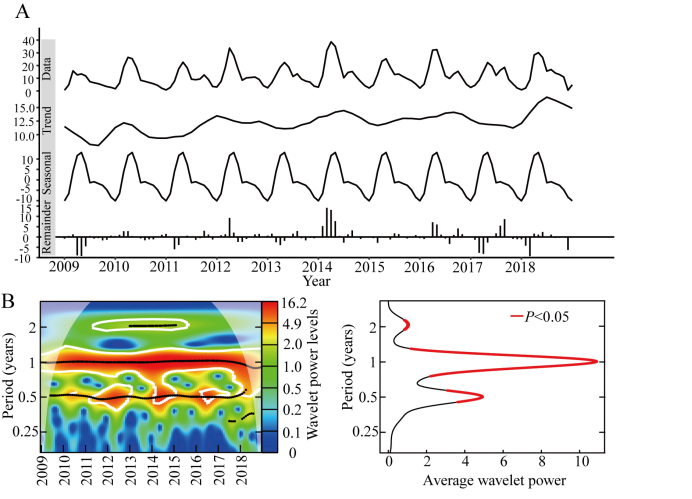


# Fig A. Seasonality and periodicity of HFMD cases in China during 2009-2018.

(A) Time series decomposition of HFMD cases including original series isolated trend seasonal and irregular components. (B) Wavelet spectrum of HFMD cases.


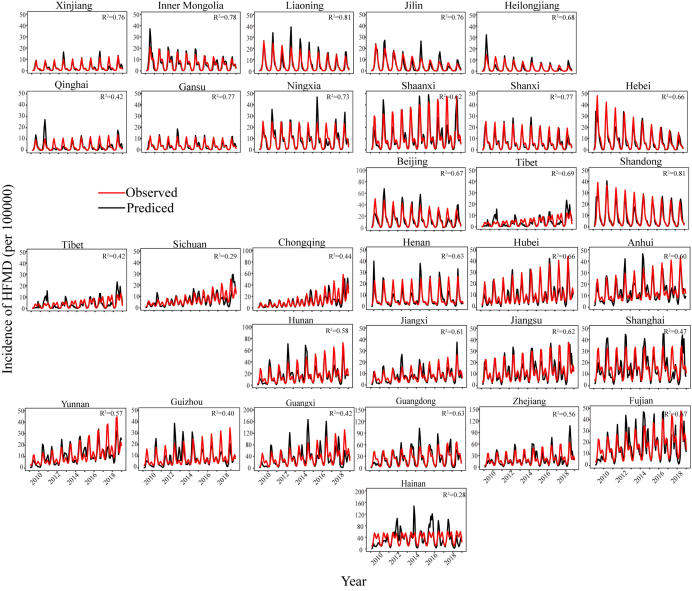


# Fig B. Fit of seasonal models in 31 provinces in China.

The seasonal model was based on a linear regression with harmonic terms for annual and semi-annual periodicities. Black curve: observed incidence; red curve: seasonal model. Provinces were ordered by their geographical location.

**
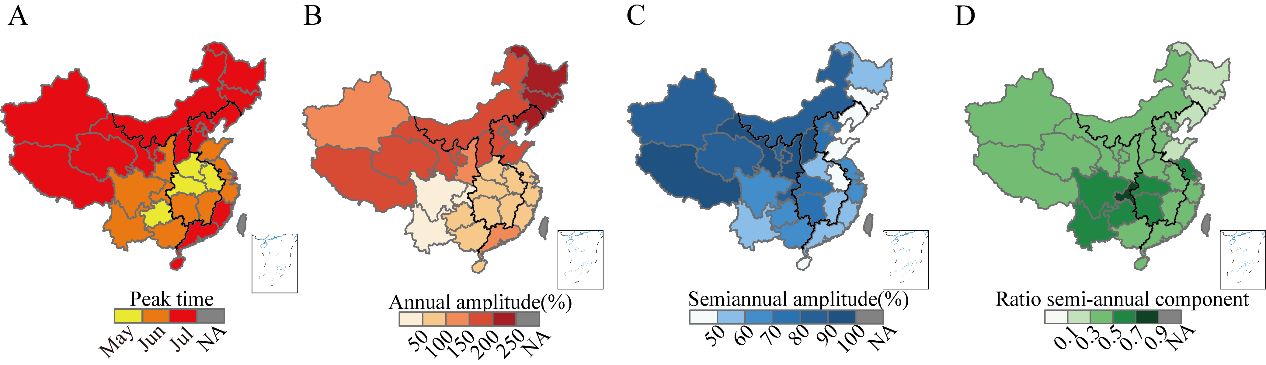
**

# Fig C. Seasonal estimates of HFMD in China during 2009-2018.

(A) Peak time. (B) Annual amplitude. (C) Semiannual amplitude. (D) Ratio semiannual periodicity. The image is generated in Arcgis Pro version 2.5 (ESRI Inc., Redlands, CA, USA), using a freely downloaded shapefile from the National Geomatics Center of China (https://www.ngcc.cn/ngcc/html/1/).

# Spatial analyses

# Table B. Global spatial autocorrelation analysis.

| Year | Moran’ s I | E(I) | S.E. | Z-score | P |
| --- | --- | --- | --- | --- | --- |
| 2009 | 0.608 | -0.033 | 0.120 | 5.341 | <0.001 |
| 2010 | 0.416 | -0.033 | 0.118 | 3.819 | <0.001 |
| 2011 | 0.433 | -0.033 | 0.096 | 4.832 | <0.001 |
| 2012 | 0.507 | -0.033 | 0.112 | 4.842 | <0.001 |
| 2013 | 0.493 | -0.033 | 0.107 | 4.913 | <0.001 |
| 2014 | 0.497 | -0.033 | 0.109 | 4.870 | <0.001 |
| 2015 | 0.436 | -0.033 | 0.096 | 4.880 | <0.001 |
| 2016 | 0.512 | -0.033 | 0.112 | 4.866 | <0.001 |
| 2017 | 0.565 | -0.033 | 0.109 | 5.502 | <0.001 |
| 2018 | 0.530 | -0.033 | 0.117 | 4.800 | <0.001 |

The values of Moran's I index range from -1 to 1. Positive and negative index values indicate positive and negative spatial autocorrelation respectively. *P* value > 0.05 indicate that the disease is randomly distributed.


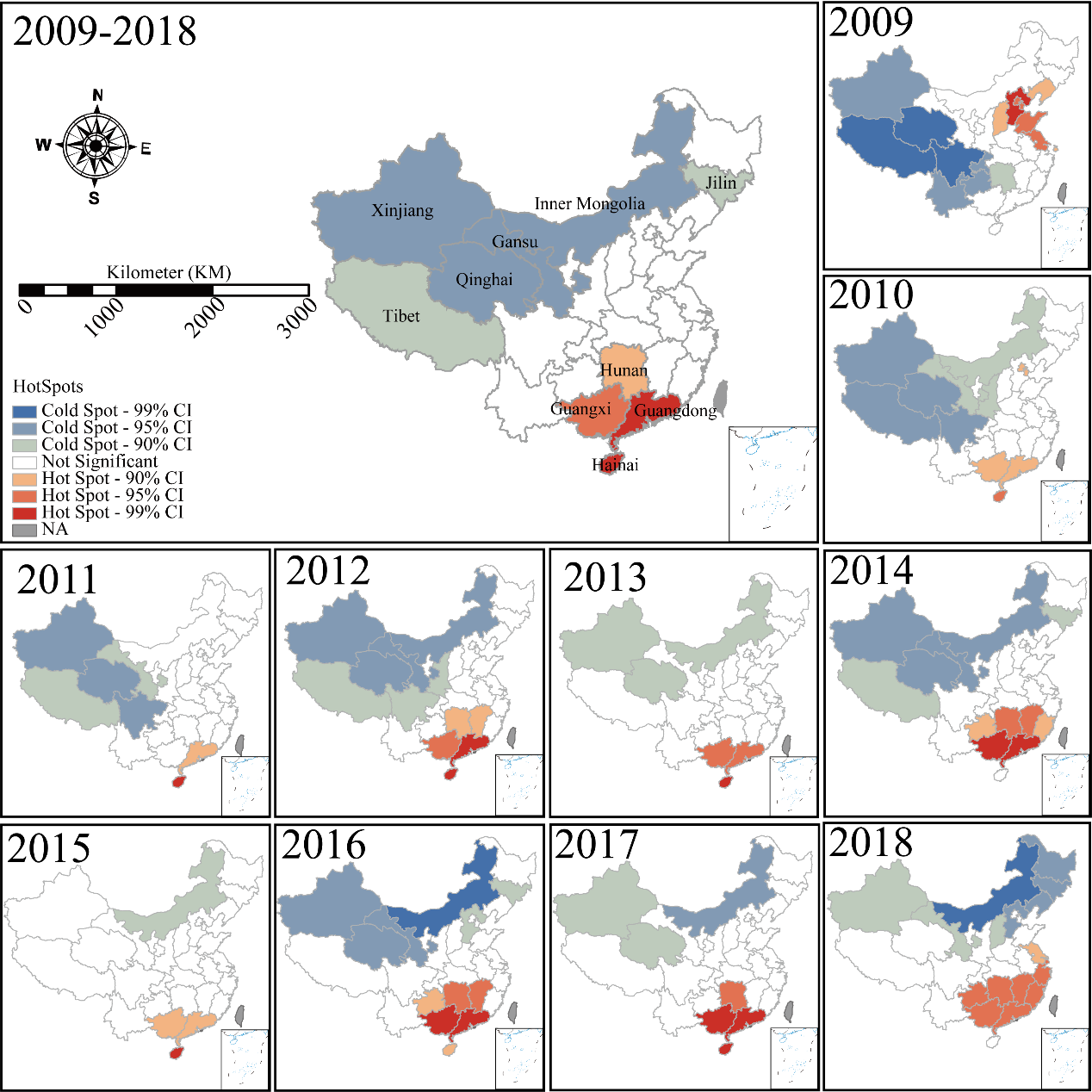


# **Fig D. Local hotspot clusters of the annual incidence of HFMD in China during 2009-2018.**

The image is generated in Arcgis Pro version 2.5 (ESRI Inc., Redlands, CA, USA), using a freely downloaded shapefile from the National Geomatics Center of China (https://www.ngcc.cn/ngcc/html/1/).

# Space-time scan analyses

# Table C. Spatiotemporal clustering of HFMD incidents in China during 2009-2018.

| Year | Cluster type | Location center | Cluster areas | Radius (Km) | Cluster month | RR | LLR | P for LLR |
| --- | --- | --- | --- | --- | --- | --- | --- | --- |
| 2009-2018 | 1 | Guangdong | 4 | 562.77 | 2013/4-2017/10 | 3.06 | 1636120.38 | <0.001 |
|  | 2 | Shandong | 12 | 750.72 | 2014/4-2014/7 | 2.01 | 188573.57 | <0.001 |
| 2009 | 1 | Heilongjiang | 15 | 1845.23 | 4-8 | 3.40 | 199095.43 | <0.001 |
|  | 2 | Hainan | 7 | 1077.18 | 4-6 | 1.73 | 15651.50 | <0.001 |
| 2010 | 1 | Guangdong | 11 | 1046.86 | 4-7 | 3.42 | 265951.58 | <0.001 |
| 2011 | 1 | Guangdong | 6 | 693.81 | 5-10 | 3.32 | 216451.88 | <0.001 |
|  | 2 | Tianjin | 16 | 1021.44 | 5-7 | 1.93 | 51504.24 | <0.001 |
| 2012 | 1 | Guangdong | 11 | 1046.86 | 4-6 | 4.11 | 388698.99 | <0.001 |
| 2013 | 1 | Guangdong | 3 | 511.50 | 5-10 | 5.74 | 400811.41 | <0.001 |
|  | 2 | Shanghai | 13 | 1040.17 | 5-7 | 1.68 | 51533.47 | <0.001 |
| 2014 | 1 | Guangdong | 11 | 1046.86 | 4-7 | 4.19 | 594815.41 | <0.001 |
| 2015 | 1 | Guangdong | 3 | 511.50 | 5-10 | 5.47 | 4006691.95 | <0.001 |
|  | 2 | Zhejiang | 9 | 763.17 | 5-6 | 2.40 | 74954.77 | <0.001 |
| 2016 | 1 | Guangdong | 12 | 1109.01 | 4-7 | 4.13 | 527019.45 | <0.001 |
| 2017 | 1 | Guangdong | 4 | 562.77 | 5-10 | 6.21 | 585418.24 | <0.001 |
|  | 2 | Shandong | 12 | 750.72 | 6-7 | 1.39 | 7770.00 | <0.001 |
| 2018 | 1 | Guangdong | 12 | 1109.01 | 5-10 | 3.58 | 456603.66 | <0.001 |

LLR log-likelihood ratio.

The *P*-value of LLR was estimated through 9999 Monte Carlo simulations.

# Spatiotemporal modeling and analyses

# Table D. Province-level potential explanatory variables of regional HFMD in China: EC1-EC8 denote environmental factors and SC1-SC10 denote socioeconomic factors.

| **Identifier** | **Environmental Variables** | **Identifier** | **Socioeconomic Variables** |
| --- | --- | --- | --- |
| EC1 | Monthly average temperature (℃) | SC1 | Population density (person/km^2^) |
| EC2 | Monthly average relative humidity (%) | SC2 | Birth rate (‰) |
| EC3 | Monthly average wind speed (m/s) | SC3 | Proportion of pupils (%) |
| EC4 | Monthly cumulative sunshine hours (h) | SC4 | Percentage under 14 years (%) |
| EC5 | Monthly cumulative precipitation (mm) | SC5 | real GDP per capita ($US) |
| EC6 | Monthly average atmospheric pressure (N/m^2^) | SC6 | Urbanization rate (%) |
| EC7 | Elevation (m) | SC7 | Passenger volume (per 10000) |
| EC8 | Monthly normalized difference vegetation index (NDVI) | SC8 | Certified doctors (per 10000) |
|  |  | SC9 | Beds of medical institutions (per 10000) |
|  |  | SC10 | School vacation |


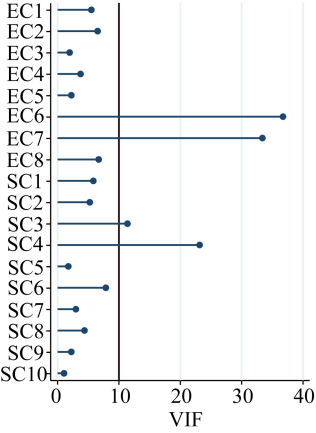


# Fig E. Variables screening procedure: remove variables with higher multicollinearity (VIF > 10).

EC1-8 and SC1-10 denote eight environmental covariates and ten socioeconomic covariates in Table D.

# Table E. BMA model selection.

| **Covariate** | **Identifier** | **Model 1** | **Model 2** |
| --- | --- | --- | --- |
| Temperature | EC1 | **6.774e-02** | 6.999e-02 |
| Relative humidity | EC2 | **2.135e-02** | 2.043e-02 |
| Wind speed | EC3 | **1.307e-01** | - |
| Sunshine hours | EC4 | **-** | - |
| Precipitation | EC5 | **-** | - |
| NDVI | EC8 | **8.976e-01** | 7.433e-01 |
| Log (Population density) | SC1 | **1.314e-01** | 1.398e-01 |
| Birth | SC2 | **5.673e-02** | 5.309e-02 |
| Log (real GDP per capita) | SC5 | **4.459e-01** | 5.101e-01 |
| Urbanization rate | SC6 | **-** | - |
| Log (Passenger volume) | SC7 | **5.256e-01** | 5.102e-01 |
| Certified doctors | SC8 | **-** | - |
| Beds of medical institutions | SC9 | **7.626e-03** | 6.736e-03 |
| School vacation | SC10 | **-7.605e-01** | -7.667e-01 |
| BIC |  | **-2.730e+04** | -2.730e+04 |
| PMP |  | **0.639** | 0.361 |

BMA Bayesian model averaging; NDVI normalized difference vegetation index; BIC Bayesian Information Criterion; PMP posterior model probability


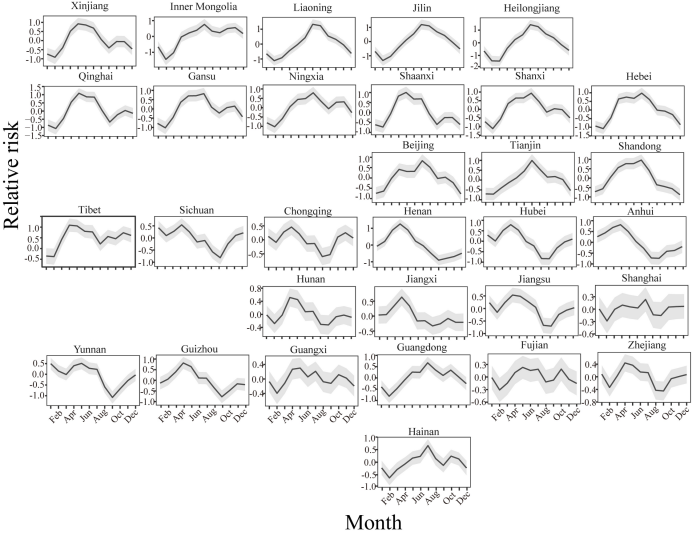


# Fig F. Contribution of the province-specific monthly random effects to HFMD incidence rate (IR) estimates.

Marginal posterior distribution of the autocorrelated monthly random effects (e.g. the annual cycle) at the linear predictor scale from January to December for the 31 provinces in China. Lines and shading areas denote posterior mean and 95% Bayesian credible interval. Provinces are ordered by their geographical location.


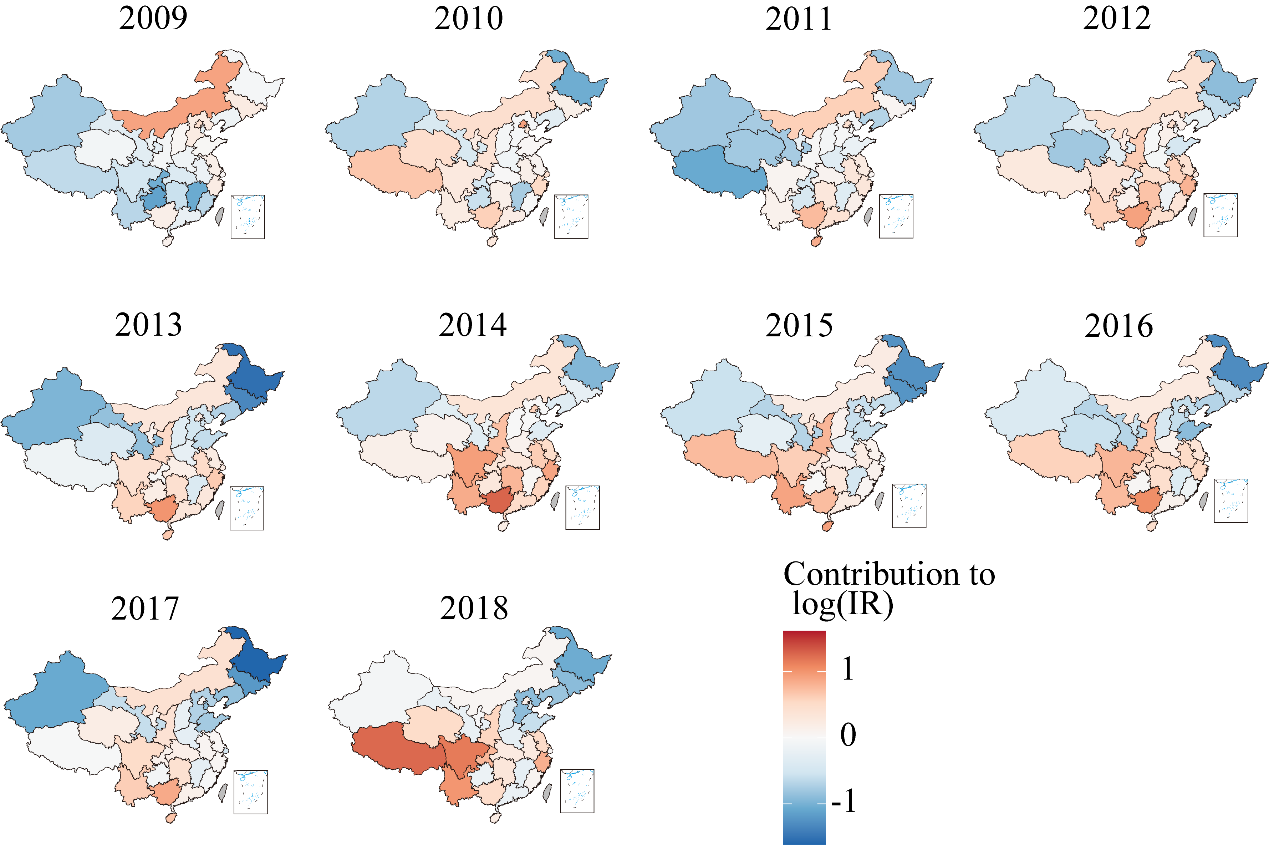


# Fig G. Contribution of year-specific spatial random effects to HFMD incidence rate (IR) estimates.

Marginal posterior mean of the combined spatially structured and unstructured random effects at the linear predictor scale per year during 2009-2018. The image is generated in Arcgis Pro version 2.5 (ESRI Inc., Redlands, CA, USA), using a freely downloaded shapefile from the National Geomatics Center of China (https://www.ngcc.cn/ngcc/html/1/).


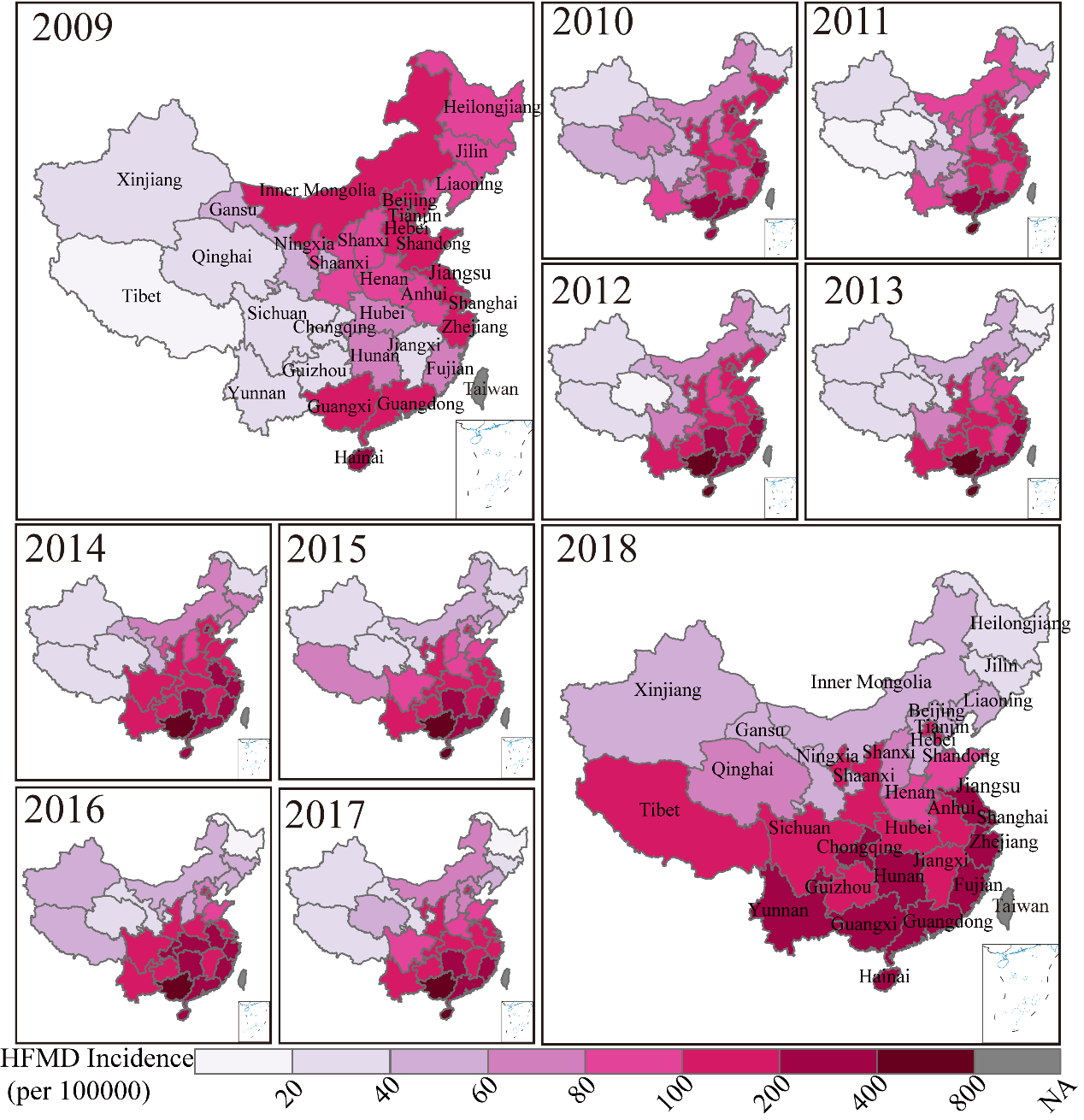


# Fig H. Posterior predictive mean HFMD incidence rate 2009-2018.

Posterior predictive mean HFMD incidence rate (IR) per 100000 people per year from 2009 to 2018 for the 31 provinces in China simulated from the hierarchical Bayesian models (refitted 12 × 10 times leaving out one month per year at a time). The image is generated in Arcgis Pro version 2.5 (ESRI Inc., Redlands, CA, USA), using a freely downloaded shapefile from the National Geomatics Center of China (https://www.ngcc.cn/ngcc/html/1/).
